# interFLOW: maximum flow framework for the identification of factors mediating the signaling convergence of multiple receptors

**DOI:** 10.1038/s41540-024-00391-z

**Published:** 2024-06-10

**Authors:** Ron Sheinin, Koren Salomon, Eilam Yeini, Shai Dulberg, Ayelet Kaminitz, Ronit Satchi-Fainaro, Roded Sharan, Asaf Madi

**Affiliations:** 1 Blavatnik School of Computer Science, Tel Aviv University, Tel Aviv, 6997801 Israel; 2 Department of Physiology and Pharmacology, Faculty of Medicine, Tel Aviv University, Tel Aviv, 6997801 Israel; 3 Department of Pathology, Faculty of Medicine, Tel Aviv University, Tel Aviv, 6997801 Israel; 4 Sagol School of Neurosciences, Tel Aviv University, Tel Aviv, 6997801 Israel

**Keywords:** Immunology, Software, Cancer

## Abstract

Cell-cell crosstalk involves simultaneous interactions of multiple receptors and ligands, followed by downstream signaling cascades working through receptors converging at dominant transcription factors, which then integrate and propagate multiple signals into a cellular response. Single-cell RNAseq of multiple cell subsets isolated from a defined microenvironment provides us with a unique opportunity to learn about such interactions reflected in their gene expression levels. We developed the interFLOW framework to map the potential ligand-receptor interactions between different cell subsets based on a maximum flow computation in a network of protein-protein interactions (PPIs). The maximum flow approach further allows characterization of the intracellular downstream signal transduction from differentially expressed receptors towards dominant transcription factors, therefore, enabling the association between a set of receptors and their downstream activated pathways. Importantly, we were able to identify key transcription factors toward which the convergence of multiple receptor signaling pathways occurs. These identified factors have a unique role in the integration and propagation of signaling following specific cell-cell interactions.

## Introduction

Cellular microenvironments consist of a complex, heterogeneous assembly of cells. The interactions or ‘crosstalk’ among these cells often dictate the cellular response. Single-cell RNA sequencing (scRNAseq) technology offers a glimpse into the transcriptional heterogeneity characterizing these microenvironments.

Central to cell-to-cell interactions is the activation of receptors by their corresponding ligands. Identifying such interactions using standard transcriptomic data is challenging. As a result, heuristic methods have emerged to estimate an “interaction potential” between cell populations. Although many efforts have been directed at this challenge^1^, the absence of a clear gold standard complicates evaluations and comparisons.

Broadly, current methods fall into two categories: those that predict cell-cell interactions solely based on ligand-receptor expression, and those that also detect intercellular signaling pathways, often using graph-based approaches. For instance, NicheNet^2^ employs the Personalized PageRank (PPR) on a ligand-signaling network to compute downstream gene activation scores. In contrast, methods like CellChat^3^ model the likelihood of crosstalk between cell populations based on gene expression of ligand-receptor pairs. We recognize that while some cell-cell interactions might be mediated by single ligand-receptor pairs, others require simultaneous interactions involving multiple receptors and ligands. Furthermore, we hypothesize that in specific microenvironments, following an interaction, downstream signaling often converges on dominant transcription factors (TFs). These TFs integrate and relay multiple signals, culminating in a cellular response.

**Fig. 1:**
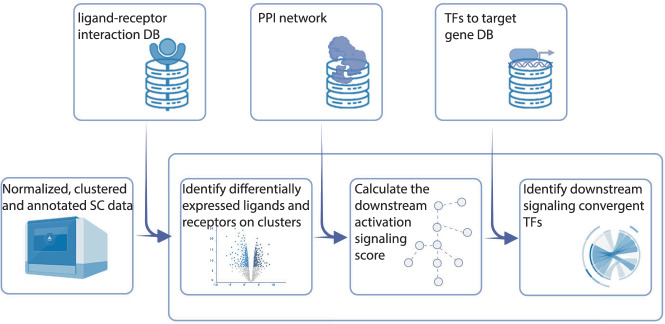
Workflow summary. interFLOW starts with a normalized, clustered, and annotated scRNAseq dataset. For each cell cluster pair, we define a “signal sender” and a “signal receiver” cluster, then identify ligands and receptors between them. For each receptor, we estimate its potential downstream signaling impact by calculating the maximum flow directed to a group of transcription factors within the Protein-Protein Interaction (PPI) network. The significance of these receptors, along with their converging transcription factors, is assessed using permutation tests. Conclusively, the algorithm determines an average interaction score between clusters, culminating in the construction of a comprehensive global interaction map for the dataset.

Building on this understanding, we introduce interFLOW—an algorithm that maps the expression of ligand/receptor pairs across cell populations using single-cell transcriptomes. Leveraging the concept of maximum flow in a protein-protein interaction (PPI) network, we further characterize the downstream pathways leading from differentially expressed (DE) receptors to dominant TFs. The maximum flow analogy aids in uncovering vital ligand-receptor pairs involved in cell-cell crosstalk. Moreover, it identifies likely signaling pathways and the central TFs aggregating signals from diverse receptors. By defining clear starting and ending points for a signaling pathway, our framework avoids assumptions regarding pathway length or confinement to a single route, enabling modeling of intricate systems like the immune response in tumor microenvironments. Applying interFLOW to a scRNAseq dataset from the GL261 glioblastoma brain tumor model^4^ —comprising both infiltrating and resident cells—we highlight the method’s capability. We unearth significant ligand-receptor pairs and spotlight key TFs orchestrating the intricate interactions between cell types (Fig. 1).

## Results

We developed a computational framework, interFLOW, that maps the expression of ligand-receptor pairs between different cell populations based on single-cell transcriptomes. interFLOW uses maximum flow computations in a PPI network to characterize the downstream pathways from a set of DE receptors toward dominant TFs. Initially, we evaluated interFLOW’s performance within a simulation framework, demonstrating the advantages of our proposed method in identifying up-regulated signaling pathways and uncovering activating receptors, while benchmarking against previous published work. Next, we present a series of analyses that showcase interFLOW’s ability to identify cell type-associated receptors and activated TFs in real datasets. Finally, we present an in-depth analysis of the communication between tumor-infiltrating CD4 + T cells and macrophages, supplemented with experimental evidence validating interFLOW’s predictions.

**Fig. 2:**
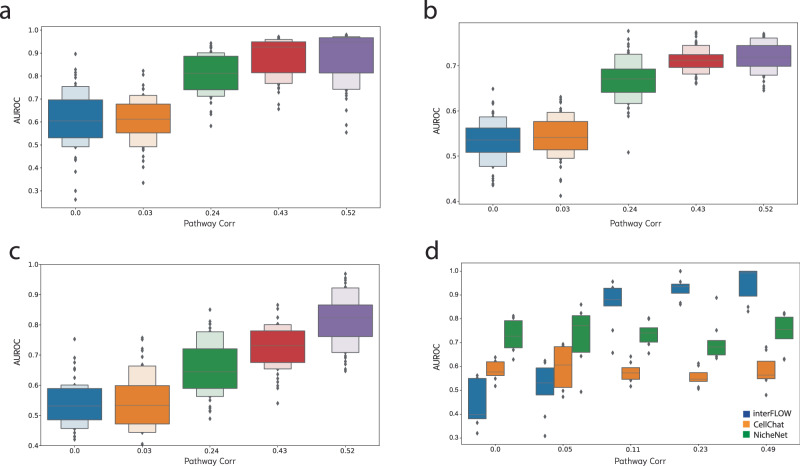
Simulation results. Across all plots, the horizontal axis represents the mean Spearman correlation of the genuine downstream activation pathway. **a** Accurate TF identification through multi-source maximum flow from all receptors to all TFs. **b** Precise identification of all genes within the authentic downstream activation pathway. **c** Effective recognition of true receptors using the FLOW score within the signal-receiving cluster. **d** Comparative assessment of interFLOW, CellChat, and NicheNet’s performance in the identification of genuine receptors within our simulation framework. In panels **a–c** the 90% and the 10% percentiles are also presented.

As a definitive ‘gold standard’ dataset for method validation is lacking, we first employed a simulation framework (methods) to assess different facets of the interFLOW pipeline. Initially, our goal was to verify whether our maximum flow approach could effectively identify the key TFs within the dataset. To accomplish this, we simulated solely the signal-receiving cluster, featuring 10 activated TFs and 25 receptors within the downstream activation pathway. Subsequently, we executed multi-source maximum flow calculations from all receptors to the TFs, calculating the area under the receiver operating characteristic (AUROC) for the activated TFs compared to all others (Fig. 2a). We conducted this analysis across different mean correlation values of the downstream activation pathways, running 50 simulations for each correlation value. As anticipated, our observations indicated that higher mean correlation values in the pathways yielded improved AUROC scores. Notably, with a mean correlation of 0.24, we achieved AUROC values exceeding 0.8. This outcome substantiates the effectiveness of the maximum flow approach in revealing the genuinely activated TFs following intercellular communication. These results provide strong evidence that interFLOW is relatively resilient to random correlations of false edges. Despite 5% of all false edges having a correlation of 0.2 or higher, we still observe adequate performance.

Next, we sought to determine if a correlation of around 0.2 is relevant in real scRNA-seq datasets. Using our GBM mouse dataset, we calculated the Spearman correlation for genes within specific protein-protein interaction (PPI) edges that are part of known KEGG pathways relevant to specific cell populations. In CD8 cells, we analyzed the genes in the “T-cell receptor signaling” pathway and found an average Spearman correlation of 0.42. Similarly, the “NF-kappa B signaling” pathway showed a correlation of 0.44. For the macrophage population, we examined the “NOD-like receptor signaling” and the “Toll-like receptor signaling” pathways, with average correlations of 0.22 and 0.23, respectively. Finally, within the tumor cluster, the “pathways in cancer” yielded a correlation of 0.15. As a reference, we examined the mean random correlation, which was 0.043.

Next, we applied the same configuration to validate the identification of all genes within the downstream activation pathways. Again, we observed that higher mean correlation values correlated with superior results. However, when considering the identification of all genes within the pathways, we observed relatively lower performance, with the maximum AUROC reaching approximately 0.7 (Fig. 2b). This discrepancy suggests that the network’s topology may limit the flow propagation in certain parts of the downstream activation pathways, resulting in some pathways remaining unidentified. Consequently, our approach exhibits high precision (above 0.9) but lower recall (around 0.4) in this context.

Finally, we assessed interFLOW’s capacity to accurately identify true receptors, comparing its performance against the CellChat (version 1.6.1) and NicheNet (version 1.1.1) methods (Fig. 2c, d). For this evaluation, we simulated two distinct clusters wherein the selected receptors and their corresponding ligands exhibited DE between the signal sender and signal receiver clusters. Simultaneously, all other genes underwent alteration by random noise with a mean of 0, ensuring the inclusion of randomly DE genes, including both erroneous receptors and ligands. For this benchmark, we updated all prior PPI networks and ligand-receptor databases to be consistent with NicheNet. Our observations consistently reveal that interFLOW achieves notable outcomes, with AUROC values above 0.9. Conversely, other methodologies such as CellChat, which does not compute downstream activation scores, or NicheNet, which does not calculate weights for the downstream activation pathway, yield results around 0.6 and 0.75 respectively within our simulation framework. It is important to note that our simulation framework operates under the assumption that activated pathways typically exhibit co-expression. Thus, in this setting, we do not expect to see improvement in performance for methods that do not compute downstream activation scores.

In each validation scenario, we compared the identified active set against a randomly sampled negative set of similar size to maintain balance in our classification problem. To mitigate any potential bias from the random sampling of positive and negative sets, we conducted each simulation 50 times, maintaining the same settings across runs.

**Fig. 3:**
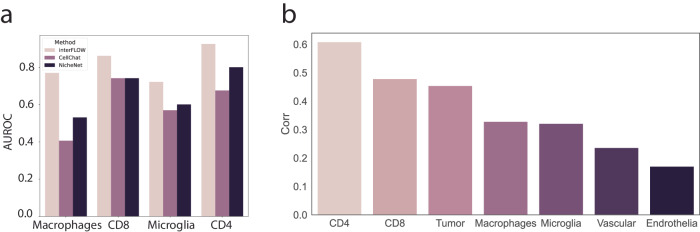
Validation of converging receptors and TFs. **a** AUROC of different methods tasked with the identification cell-type specific receptors. **b** Spearman correlation between TFs flow score normalized by node degree and the gene target enrichment score per cell type.

Next, to evaluate our framework, interFLOW, using real data, we performed systematic analyses at each step using a single-cell RNA-seq dataset from the GL261 murine brain tumor model^4^. Initially, we compared the ability of interFLOW, CellChat, and NicheNet to identify cell-type-associated receptors. To this end, we curated an unbiased dataset containing known cell-type-associated receptors (CD8, CD4, Macrophages, and Microglia) and a combined set of negative receptors (B cells, Mast cells). The full list used for this benchmark is presented in Supplementary Table 1. Our methodology involved calculating the AUROC for each cell population, assessing the method’s ability to accurately identify cell-type-specific interactions compared to the negative set (Fig. 3a). As we expect that receptors known to be associated with specific cell types will generally be more active than those that are not. We observed that interFLOW presents higher performance relative to previous methods across all tested cell types, achieving an area under the curve (AUC) of around 0.8 across all cell types, with particularly notable performance in CD4 T cells, achieving an AUC of 0.93.

### Validation of identified transcription factors

We hypothesized that the intracellular signals from multiple interacting receptors should flow towards a layer of transcription factors, which in turn will propagate the signaling into a cellular response. We further assume that enrichment of the TF target gene expression strongly indicates TF activation. To investigate this, we compared the -log (Targets Enrichment *P* value) and the normalized flow score of all TFs in each cell type. However, without normalization, the importance value of each TF was strongly associated with the node’s degree of centrality in the PPI network. Therefore, we normalized the flow value by its centrality. We found a Spearman correlation of 0.4-0.64 for each of the immune cell populations (Fig. 3b). This suggests that our flow score provides some indication for the activation of a given transcription factor.

There was a relatively high variance in performance quality across different cell populations (Fig. 3b). Consequently, we performed an in-depth analysis of the low-scoring endothelial cell population, which averaged around 0.07, compared to 0.3 in the CD4 cells. This suggests that endothelial cells may not undergo significant interactions in the current biological settings, which could result in lower scores.

### Validation of downstream gene scores against MSigDB

To validate our downstream gene scores, we utilized the MSigDB^5^ immune signature database (C7). This database contains over 5000 gene signatures associated with various cell types and conditions. We carefully curated a dataset of signatures regulated in each cell type, ensuring the inclusion of only up-regulated signatures for each cell type while deliberately excluding gene sets that compared the same cell types under different conditions.

We performed an enticement analysis of the genes with significant flow values against the curated subset of the C7 database. To this end, we first performed a permutation test (methods) for the amount of flow going into each node in the network, nodes/genes with significant flow values were used to perform an enrichment test for each gene signature. Next, we used Fisher Exact Test to examine if the proportion of the gene signatures that were returned from the analysis and are associated with the correct cell types is significantly greater than the overall proportion of the cell type signatures in the database.

**Fig. 4:**
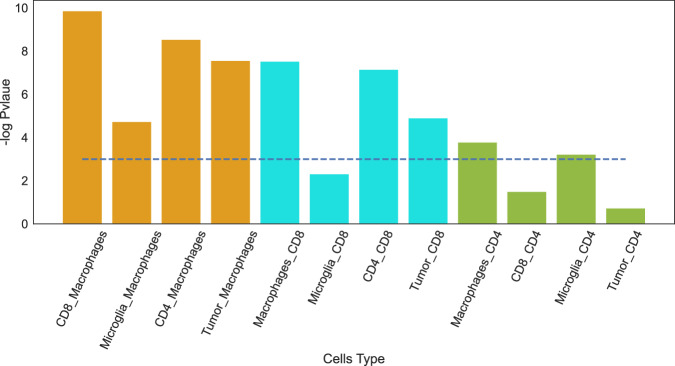
Validation against MSigDB C7 immunological signature database. interFLOW was applied to the interaction between each two cell types, and a signature containing significant genes in the receiving cell type was defined. Enrichment of each gene signature associated with the correct corresponding cell type from the MSigDB C7 immunological database was calculated. The blue line represents the significant threshold as -log(0.05) using Fisher exact test.

Indeed, the proportion of the gene signatures associated with the correct cell types is, in most cases, significantly larger than their proportion in the entire database (Fig. 4). Thus, there is an association between genes with significant flow value and biological function in a given cell type. We illustrated a similar analysis conducted using NicheNet, which demonstrated a comparatively lower accuracy in associating the correct cell type (Supplementary Fig. 4).

### Robustness of interFLOW

**Fig. 5:**
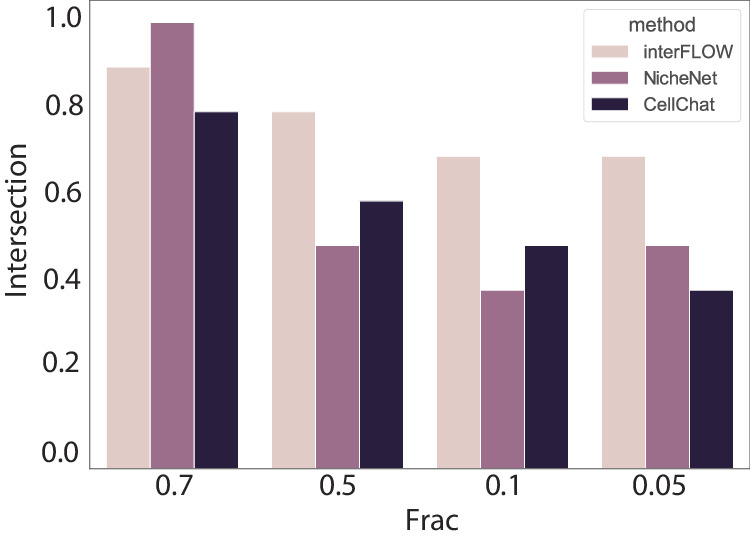
Robustness evaluation. Intersection between the 10 highest predictions in the full dataset to the highest prediction of the sampled dataset at different fractions.

We evaluated the robustness of interFLOW and further compared it to CellChat and NicheNet by interactively sampling the data set in fractions of 0.7, 0.5, 0.1, and 0.05. For each method, we calculated the intersection of the top 10 predictions from each fraction to the top 10 predictions for the full dataset. interFLOW exhibited exceptional robustness, outperforming both NicheNet and CellChat across most data fractions tested. Notably, while NicheNet showed superior performance over interFLOW at higher data fractions, interFLOW consistently demonstrated the best performance when the dataset was reduced to less than 50% of its original size (Fig. 5).

A further in-depth comparison between interFLOW results and CellChat across multiple cell types on the GBM dataset shows that overlap predictions between the methods resulted in higher DSA scores overall **(**Supplementary Fig. 5). Furthermore, we applied interFLOW with the presented validation framework to an additional single-cell RNA seq dataset of Glioma brain tumor^6^ (Supplementary Fig. 6).

### In-depth analysis of Macrophages - CD4 + T cell interaction

After establishing the accuracy of our method, we investigated specific cell-cell interactions inferred from the brain tumor dataset. As was previously shown, increased infiltration of T cells is associated with prolonged survival of GBM patients^7^ and CD4 + T cells play a key role in coordinating antigen-specific immunity through their high plasticity and cytokine-producing ability^8^. However, tumor-associated macrophages (TAMs) have been associated with high-grade gliomas and a worsened outcome. It has been proposed that they produce cytokines and other factors to promote a tumor-supportive environment by suppressing the proliferation of anti-tumor CD4+ and CD8 + T cells and promoting the activity of regulatory CD4 + T cells^9^. In fact, we have previously identified increased infiltration of CD4 T cells following perturbation of the P-selectin/P-selectin ligand-1 (SELP/PSGL-1) axis. Interfering with the crosstalk between pro-tumorigenic macrophages and CD4 + T cells has been proposed as a therapeutic strategy; however, detailed information about this interaction is lacking. Thus, out of the multiple cell-cell interactions detected by our method, we used interFLOW to specifically investigate the interaction between macrophages and CD4 + T cells in the SELP-knockdown-GL261 murine glioblastoma brain tumor model.

**Fig. 6:**
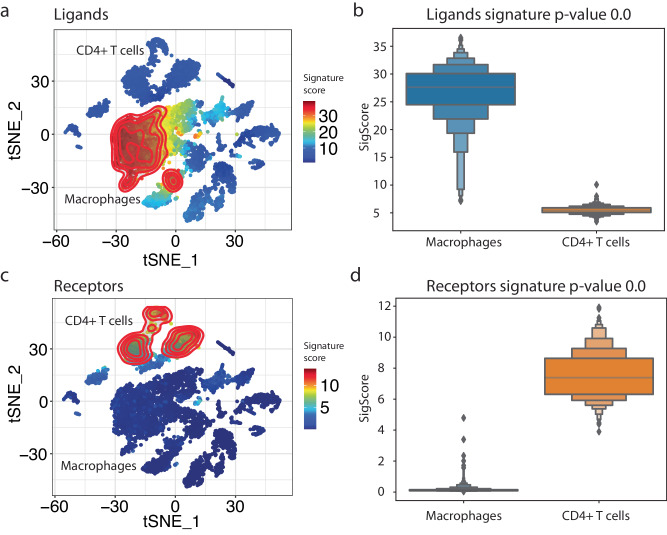
Identification of potential ligand-receptor pairs between macrophage and CD4 + T cell clusters. **a** Signature projection of identified receptors on the CD4 + T cell projected on the tSNE space. **b** Receptors signature distribution and Wilcoxon Rank-Sum *p*-value results. **c** Signature projection of identified ligands on the macrophage cells. **d** Ligands signature distribution and Wilcoxon Rank-Sum *p*-value results. The bar plots were generated as letter-value plots, better suited for dispelling larger datasets, presenting the following percentiles: 6.25, 12.5, 18.75, 25 (Q1), 31.25, 37.5, 50 (Q2), 62.5, 68.75, 75 (Q3), 81.25, 87.5.

interFLOW identified 37 ligands and 39 corresponding receptors, altogether forming 102 DE ligand-receptor interactions. To visually demonstrate the ligands and receptors detected by our analysis, we projected their signature scores onto the tSNE reduction plot (Fig. 6). As expected, the signal-receiving cluster was strongly associated with the receptors, while the signal-sender cluster was associated with the corresponding ligands.

**Fig. 7:**
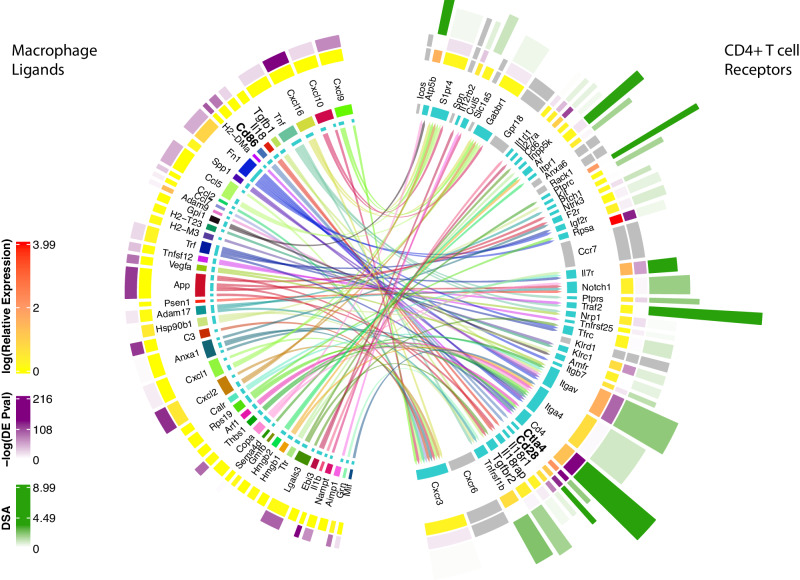
Ligand-receptor interaction with integrated receptor downstream activation signaling score. Detailed analysis of the differentially enriched ligand-receptor interactions between macrophage (signal-sender) and CD4 + T cell (signal-receiving) clusters. Inner lines indicate potential ligand-receptor connections and the width of the inner circle ribbon indicates the number of potential connections. The second circle ribbon reflects the expression level, the third outer ribbon indicates the Wilcoxon Rank-Sum *p*-value of upregulation compared to all other clusters in the dataset and the outer ribbon indicates the downstream activation score (DSA). Eight receptors that did not show significant value in the permutation test are colored in grey.

Following the identification of ligand-receptor pairs and further calculation of the DSA for each receptor, interFLOW generates a visualization plot that can be further examined for specific interaction between the two clusters. This plot provides the following information: the many-to-many ligand-receptor potential interactions, expression level, the significance of differential expression, indication for the downstream signal activation and its statistical significance (Fig. 7).

Out of these multiple significant ligand-receptor interactions, here we focus on two opposite potential interactions between macrophages and CD4 T cells, which further highlights the complexity of signals a cell receives in this microenvironment. On the one hand, a pro-inflammatory interaction through the IL18 receptor and its ligand IL18a, leads to type I activation in the form of IFNγ synthesis from Th1 cells^10^. On the other hand, an immunosuppressive interaction through the TGF-β receptor and its ligand TGF-β. Moreover, signaling via TGF-β can subvert T-cell immunity by favoring regulatory T-cell differentiation, further reinforcing immunosuppression within tumor microenvironments^11^. Another example of the complexity of this crosstalk between these two cell types is the two receptors co-stimulatory CD28^12^ and co-inhibitory CTLA4 which compete for the same ligand CD86.

### Signaling converging transcription factors in CD4 + T cells following interaction with macrophages

**Fig. 8:**
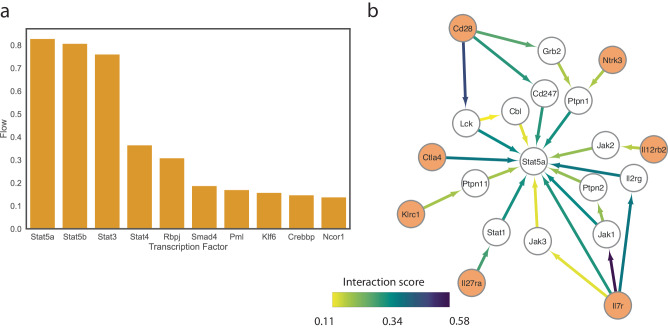
Identification of downstream signaling converging transcription factors for CD4 T cells. **a** A bar plot showing the top-ranking TFs in the interaction between macrophages and CD4 + T cell clusters shown in Fig. 7. **b** Subnetwork of the flow from multiple receptors (orange) to Stat5 transcription factor. The edge color and width represent the amount of flow that is passing through the edge as a proxy for the significance of the pathway in the subnetwork.

As part of the framework, interFLOW highlights significant TFs that are likely to be activated by the signals received from multiple receptors. To this end, we applied the multi-source max flow algorithm from all the receptors down to the defined set of transcription factors and calculated the amount of flow that goes through each transcription factor. Next, to rank the contribution of the different TFs, we calculated the importance coefficient of the TF (Fig. 8a). Specifically, we removed each of the TFs from the network, and recalculated the multi-sourced max flow in order to obtain the difference in the maximum flow in the network. This yielded a subnetwork that represents the flow pathways from multiple receptors toward a specific TF, allowing us to further investigate a specific signal transduction pathway and detect potential hubs (Fig. 8b).

The top-ranking transcription factor, Stat5, was found to play an important role in the differentiation of various T helper type 1 (TH1), TH2, TH9, T helper type GM-CSF (TH GM), and Treg cell subsets. In B-cell lymphoma tumor models persistent STAT5 activation reprograms the epigenetic landscape in CD4 + T cells to drive polyfunctionality and antitumor immunity^6^. Another TF that was highly ranked was Stat3 which in many cases opposes Stat5 in differentiation^13^. While the importance of the identified TFs to the survival, differentiation and cytotoxic capacity has been previously demonstrated, here we show that these TFs play a role in the specific interaction between macrophages and CD4+ tumor-infiltrating lymphocytes (TILs) in the context of the brain tumor microenvironment^14^.

**Fig. 9:**
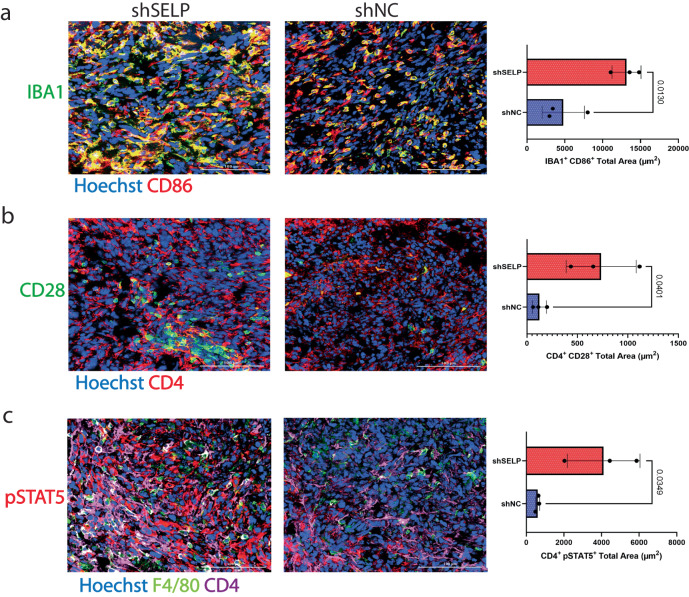
Silencing SELP in GL261 glioblastoma tumors alters the macrophages’ co-stimulation of CD4 T cells. Immunostaining analysis of GL261 glioblastoma tumors showed increased expression of CD86 in macrophages (**a**) and higher levels of CD28 and pSTAT5 in CD4 + T cells (**b, c**) in SELP knockdown GL261 tumors (shSELP) compared to the negative control (shNC). For all panels, data are represented as the mean ± s.d. Each dot (*N* = 3) indicates the average of five fields in the tissue. The analysis was carried out using an unpaired two-tailed T-test.

Next, we aimed to demonstrate that the predictions generated by interFLOW can effectively capture interactions that are specifically associated with a distinct biological context. We have previously shown that bone marrow-derived macrophages, the silencing of SELP in murine GL261 glioblastoma cells induced a more anti-tumorigenic phenotype of the Bone marrow-derived macrophages (DMDM) and additional alterations in the tumor microenvironment including increased CD4 + T cell infiltration and activation^4^. Our objective was to highlight changes in factors involved in the macrophage and CD4 + T cell interaction following SELP silencing. To achieve this, we conducted immunofluorescence staining of selected potential candidates on frozen section slides (Fig. 9, Methods). To investigate the phenotypic alterations in CD4 + T cells, and their potential interactions with macrophages within the tumors, we first analyzed the expression level of CD86 on macrophages by performing co-immunostaining for IBA1 and CD86 (Fig. 9a). Among the IBA1^+^ population, we noted an elevated proportion of cells co-expressing CD86, suggesting increased microglial/macrophages activation (Fig. 9a).

CD86 on macrophages was predicted to interact with CD28 on tumor infiltrating CD4 + T cells. To corroborate this prediction, we first examined the expression of CD28 on CD4 + T cells by conducting co-immunostaining for CD4 and CD28. As expected, we indeed observed elevated levels of CD28 expression in CD4 + T cells following SELP knockdown (Fig. 9b). Furthermore, our predictions indicated that intracellular signaling initiated from CD28, along with multiple other receptors, would converge toward the transcription factor STAT5 (Fig. 8). In line with these predictions, we conducted co-immunostaining for CD4 and STAT5 and confirmed increased STAT5 expression in CD4 + T cells after SELP knockdown (Supp. Figure 7a). Moreover, we identified a positive correlation of approximately 0.3 between the expression levels of CD28 and STAT5 across different slides, suggesting co-expression between the receptor and its downstream transcription factor. As the downstream functionality of STAT5 depends on its phosphorylation, we further stained the cells for pSTAT5 and confirmed its increased expression in CD4 + T cells after SELP knockdown (Fig. 9c). However, overall, we found only a slight negative correlation between the levels of pSTAT5 on CD4 + T cells and their distance from macrophages, which could be attributed to the dynamic nature of these cells (Supp. Figure 7b). Together, these results demonstrate the validity of our predicted cell-cell interactions at the protein level. They validate the existence of a specific and context-related ligand-receptor interaction between macrophages and CD4 + T cells in glioblastoma while also shedding light on the intracellular mechanism within CD4 + T cells following this interaction.

### Crosstalk between tumor and macrophages

BMDMs recruited to the brain under pathology interact with tumor cells via SELP/PSGL-1, contributing to glioblastoma progression. However, SELP/PSGL-1 is not the only axis of interaction between the two cell types. Thus, we used interFLOW, as shown above, to investigate the interaction between macrophages and tumor cells in the SELP-knockdown-GL261 murine glioblastoma brain tumor model.

To initiate the process, we first identified DE ligand-receptor pairs, which encompassed 37 distinct ligands and 39 corresponding receptors, collectively resulting in a total of 102 unique ligand-receptor interactions (Supp. Figure 8a).

Here, we chose to highlight the interaction between the ligand Ccl5, found on tumor cells, and its corresponding receptors, Ccr1 and Ccr5, which are present in macrophages. Notably, Ccl5 operates within the glioblastoma microenvironment through Ccr1 and Ccr5 in a redundant fashion. This redundant action is associated with the mediation of microglia/macrophage-stimulated glioma invasion, indicating that the infiltration of immune cells and their survival rates do not rely solely on the individual expression of either receptor. This is another example of the complexity of signals a cell receives in this microenvironment.

**Fig. 10:**
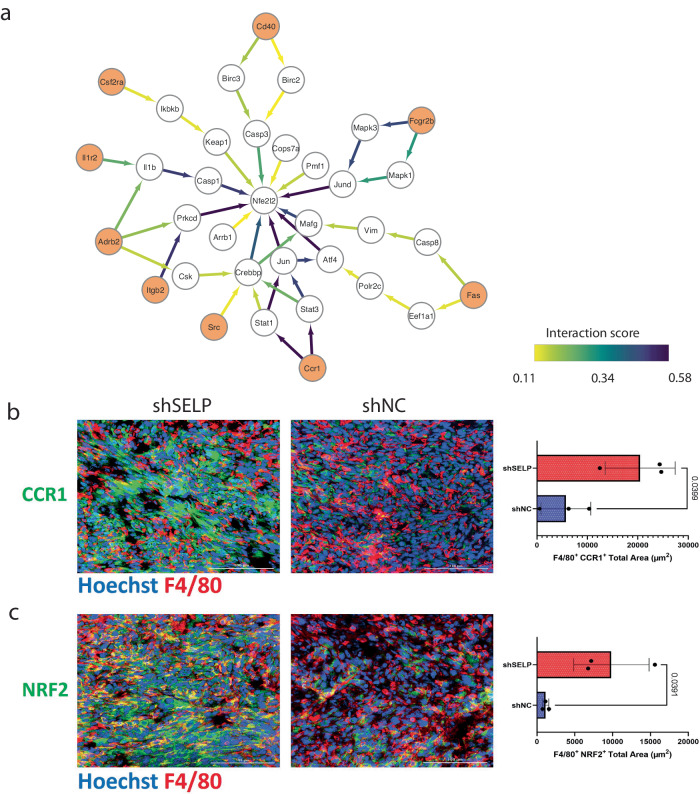
Silencing SELP in GL261 glioblastoma tumors alters macrophage activation. **a** Subnetwork of the flow from multiple receptors on macrophages (orange) to Nfe2l2 (NRF2) transcription factor. The edge color and width represent the amount of flow that is passing through the edge as a proxy for the significance of the pathway in the subnetwork. **b** Immunostaining analysis of GL261 glioblastoma tumors showed increased expression of CCR1 and (**c**) NRF2 (Nfe2l2) in macrophages in SELP-knockdown GL261 tumors (shSELP) compared to the negative control (shNC). For all panels, data are represented as the mean ± s.d. Each dot (*N* = 3) indicates the average of five fields in the tissue. The analysis was carried out using an unpaired two-tailed T-test.

Next, we identified signaling converging transcription factors in macrophages following interaction with tumor cells (Supp. Figure 8b). One of the top-ranking TFs was Nfe2l2 (NRF2), which was previously suggested to suppress macrophage inflammatory response by blocking proinflammatory cytokine transcription^15,16^ (Fig. 10a).

Experimental validation of Ccr1 expression on tumor-infiltrating macrophages, using co-immunostaining for F4/80 and CCR1, revealed a notable increase in the SELP-knockdown-GL261 murine glioblastoma brain tumor model when compared to the control group (Fig. 10b). Further validation of Nfe2l2 expression on macrophages, using co-immunostaining for F4/80 and NRF2, showed an increase upon SELP silencing (Fig. 10c). Again, we observed a positive correlation of 0.55 between the expression of CCR1 and Nfe2l2. NRF2 regulates the transcriptional activation of its target genes through various mechanisms, encompassing transcriptional, post-transcriptional, and post-translational processes. Under oxidative stress conditions, NRF2 translocates into the nucleus, but it also plays a crucial role in preserving the integrity of mitochondrial DNA^17^. Therefore, NRF2 operates in both the cytoplasm and the nucleus. Indeed, further analysis revealed an increase in NRF2 nuclear expression in macrophages following SELP knockdown (Supp. Figure 8c,d). Together, these results highlight some of the additional alterations that macrophages undergo once the SELP/PSGL-1 axis is perturbed.

### Global interactions in the tumor microenvironment

**Fig. 11:**
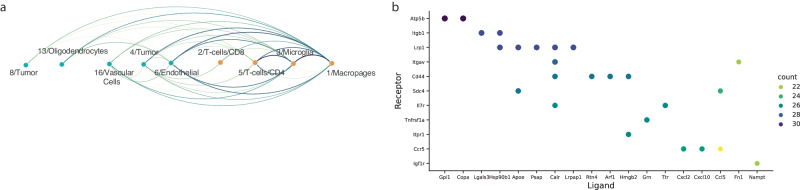
Global interaction map. **a** Global interaction plot, demonstrating interactions between the different clusters in the dataset. Edges radiate from the signal sender cluster to the signal receiving cluster, edge colors and width represent the strength of the interaction. **b** Top 25 ligand-receptor interactions that were identified as active between multiple cell types in the dataset.

By applying the previously described method on each pair of clusters in the data set, we have generated a global map of interactions between all cell populations in the dataset (Fig. 11a). We hypothesized that generating such an interaction map could help us to better understand the global dynamics between different cell populations in the data set, and uncover the key interaction axes common and cell-type specific to a studied biological system (Fig. 11b).

As can be observed in Fig. 11b, there are almost no meaningful interactions between non-immune cell populations (endothelial, vascular, tumor and oligodendrocyte) (Supp. Figure 9). However, they do show relatively strong interactions with antigen-presenting myeloid cells (macrophages and microglia). In addition, we detected expected interactions between CD4+ helper T cells and the antigen-presenting populations.

Finally, our global analysis can also be used to highlight common ligand-receptor interactions between different cell types. Indeed, we found many such interactions which are common to communications between multiple cell types.

Taken together, our global analysis was well adapted to capture some of the known cell-cell interactions in this microenvironment and highlighted unique and more common interactions that must be taken into account in perturbation applications.

## Discussion

The task of quantitative cell-to-cell interaction modeling based on transcriptomic data and single cell transcriptomics specifically is heuristic by nature. First, gene expression data is not able to detect direct interaction between different cells, which leaves us only with the detection of the interaction potential. Second, gene expression also does not necessarily reflect gene activation. Third, the relatively low mRNA capture rate of the scRNAseq technologies generates “zero-inflated” datasets, which makes it harder to detect such complex events per cell.

Here, we proposed a computational framework view of the maximum flow problem. Our working hypothesis is that a significant proportion of the signals received by the cell via its receptors will propagate downstream toward a layer of transcription factors. These TFs will in turn affect a vast number of genes and converge into a cellular response. To examine the validity and performance of our suggested framework, we compared it to two state-of-the-art methods, showing that interFLOW is both robust and biologically relevant. Importantly, we performed an end-to-end analysis of the interaction between macrophages and CD4 + T cells in glioblastoma microenvironment including experimental validations. We showed that interFLOW could uncover interesting ligand-receptor interactions in the data, and identify a set of TFs that may have an important role in regulating the functionality of CD4+ cells following such interaction in the tumor microenvironment.

More specifically, to validate the proteins identified by our algorithm involved in the interaction between macrophages and CD4 + T cells, we performed co-staining of CD4^+^ cells together with STAT5, pSTAT5 and CD28. STAT5 enhances robust expansion, infiltration, and the response of CD8 + T cells^6^. This is driven by the constitutive expression of STAT5 in CD4^+^ T cells, which leads to extensive remodeling of their transcriptional and epigenetic profile. We found that the downregulation of SELP in glioma cells results in increased expression of CD28 in CD4 + T-cells. CD28 is widely acknowledged as a co-stimulatory molecule for T-cell activation, interacting with CD80/86 on antigen-presenting cells, such as TAMs^12^. Consequently, the upregulation of CD86 in macrophages and CD28 in CD4 + T-cells implies a significant role for SELP-PSGL-1 axis in modulating the brain’s immune system. However, our analysis identified numerous ligand-receptor interactions between macrophages and CD4 + T cells converging on STAT5. Therefore, we cannot exclusively attribute the upregulation of STAT5 or pSTAT5 to the CD86-CD28 interaction, particularly considering the detection of IL7r, which has previously been shown to promote STAT5 phosphorylation^18^

However, SELP-PSGL-1 is not the only axis by which tumor cells affect macrophages. Here we highlight CCR1-NRF2 as an additional pathway that is upregulated following SELP perturbation. According to previous studies, increased levels of CCR1 are associated with increased invasion of glioma cells. Glioma-conditioned medium enhances the expression of CCR1 on TAMs, establishing an autocrine loop that facilitates the response to CCR1 ligands: CCL3, CCL5, CCL6, and CCL9^19^. The Nuclear factor erythroid 2 (NRF2)/Keap1 pathway has garnered significant interest for its role in cancer progression and glioma in particular as a TF regulating several antioxidant elements and associated with poor prognosis^16,20^. Regarding NRF2’s role as a transcription factor in redox control, it has been demonstrated that NRF2 inhibits RNA polymerase 2 binding to pro-inflammatory cytokines in macrophages, leading to a decrease in the locus activity of IL6 and IL1β^19^. Collectively, these findings suggest that a combination of a CCR1 inhibitor and a SELP inhibitor may exhibit a synergistic effect in suppressing the invasive characteristics of gliomas. Such an approach holds the potential to reshape the immune landscape and enhance patient survival rates.

To further demonstrate the power of interFLOW, we applied it to another scRNAseq obtained from an independent Glioma brain tumor mouse model containing both wild-type and myeloid MHCII knockout mice^21^. Using interFLOW, we identified multiple potential interactions between various cell types some of which were differential between the WT and the KO cells. Spp1- Itgb1 was found to be a key communication axis between macrophages and CD8 + T cells in the KO samples. Furthermore, our method identified that the downstream signaling pathways in the interaction between these two cell types converged at the transcription regulator Nfat2, which is a positive regulator of Tox (critical regulator of T cell exhaustion). Complementary biological experiments were able to validate the effect of Spp1-Nfat2-Tox axis as was predicted by our method.

Cell-cell interaction involves the activation of multiple receptors followed by complex intracellular signal transduction. These signals must be aggregated for the cell to reach a reaction decision, thus highlighting the role of key transcription factors as a layer that aggregates the different signals into a cellular response is unique to our approach. Further characterization of transcription factors identified in a specific biological setting is, however, needed. Nevertheless, this approach opens new therapeutic avenues for the perturbation of a cellular response following cell-cell interactions.

Similar to our framework, CellCall^22^ assumes that signaling via receptor leads to transcription factor activation. The CellCall model assigns an activation score for a set of KEGG pathways while also taking into account the enrichment of TF’s target genes in each pathway. Rather than looking at a set of disjoint pathways, our suggested interFLOW approach takes advantage of an interaction network. This allows the discovery of novel activation pathways related to specific cell populations and biological conditions. Furthermore, our maximum flow framework can model the underlying assumption that signals from different receptors are converging into a subset of dominant transcription factors. Lastly, our methodology could assign activation scores for transcription factors based on network importance rather than a single pathway. In this article, we have shown that the amount of flow of a given receptor is associated with its upregulation in different cell populations. The signal flow was also found to be associated with the upregulation of the transcription factor predicted to further converge the signals. Thus, our interaction potential between two clusters includes not only the expression of the receptor and ligand but its entire signal transduction pathway.

On a global scale, our framework allows us to inspect the interaction map of an entire single-cell data set. Indeed, various microenvironments are characterized by multiple cell-type populations that constantly interact with each other. Such complex and dynamic interaction as well as a comparison between different states of the system could greatly benefit from such global analysis.

It is important to note that the activation of a signaling pathway in biological systems usually also includes post-translational modifications, which are not measured by any form of RNA seq data. Thus, any activation score that is based solely on RNA expression will never capture the entire pathway activation process. Using data sets taken from the tumor microenvironment, we have shown that our method can reflect dynamics in the data that agree with known biological assumptions. To reduce the effect of the data sparsity associated with scRNAseq most of our calculations are done at the cluster level. However, we believe that our suggested framework, with the proper normalization, may enable us to assign an interaction potential score to every single cell in the dataset, and by that reveal more complex biological dynamics inside the clusters. Combining such cell-specific scores with other single cell analysis approaches will allow us to ask more complex questions, such as how the interactions can affect the cell differentiation trajectories, and understand how cell-to-cell interactions change in different biological conditions.

Recent work in the field has begun to emphasize the integration of biological replicates and different conditions into their models to reduce false predictions^23^. In our study, we presented the interFLOW framework specifically for single-dataset analysis, primarily to demonstrate the potential and advantages of our maximum flow methodology. Accounting for multi-sample gene-gene relationships in our analysis, in order to refine our predictions, remains an important direction for future work with our framework.

## Methods

### Data processing, clustering and annotation

The pipeline receives Seurat objects following data processing, normalization, clustering and annotation as previously described^24^. Briefly, the pipeline consists of the following steps. **LogNormalize**: each feature count for each cell is divided by the total counts for that cell and multiplied by a scale factor. **Dimensionality reduction:** PCA and tSNE are calculated from the scale normalized data matrix, where each feature normalized expression is scaled across the cells. The number of PCs for the clustering was manually selected based on an elbow plot showing the gain in variance with each additional vector. **Clustering:** First, we calculated the k-nearest neighbors and constructed the KNN graph, in the reduced PCA space. On that graph, a modularity score is optimized using the Leiden clustering method^25^.**Cluster annotation:** was performed manually by the use of known cell population markers and projection of known cell-type gene signatures on the tSNE plots^26^.

### Single-cell gene signature scoring

Single-cell gene signature scoring was used to emphasize the differential expression of ligands and receptors on interacting cell subtypes, as previously described^27^. Briefly, scores were computed by first sorting the normalized scaled gene expression values for each cell followed by summing up the indices (ranks) of the signature genes. A contour plot which takes into account only cells that have a signature score above the indicated threshold was added on top of the tSNE space, to further emphasize the region of high-scoring cells.

### Finding differentially expressed receptor-ligand pairs

Ligand and receptor pairs were retrieved from the CellTalkDB database^28^. As a first step, we identified receptors that are DE in the signal receiving cluster and their corresponding ligands which are DE in the signal sending cluster, using the Seurat package “FindMarkers” function. We applied the Wilcoxon Sum Rank with limit testing chosen to detect genes that display an average of at least 0.2-fold difference (log-scale) between the two groups of cells and genes that are detected in a minimum fraction of 0.2 in the upregulated group.

### Threshold optimization

The threshold for differentially expressed (DE) receptor-ligand pairs determines the initial filtering prior to in-depth analysis. This threshold may vary across datasets containing different cell populations, which can differ in their levels of similarity to one another. Generally, we prefer to choose a more inclusive threshold, as the Downstream Signaling Activity (DSA) score indicates which receptors might be significant in interactions. Thus, we typically set the threshold at either 0.1 or 0.2. A lower threshold includes a larger number of receptors for analysis, though these receptors generally have lower DSA scores. To illustrate the impact of different thresholds, Supplementary Fig. S1 displays the dynamics of the number of receptors and the mean DSA score across various thresholds.

### Identifying activated transcription factors

To pinpoint potentially activated transcription factors within each cluster, we conducted an enrichment test utilizing the Dorothea TF-gene target database^29^. For a specified gene target list, an unpaired Wilcoxon Sum Rank test was applied to compare the rank distributions of the gene list against the remaining gene expression vector. This allowed us to test whether the mean expression of genes in the list stemmed from the same distribution as the background. P-values obtained were subsequently adjusted using the FDR correction.

### Calculation of downstream activation score (DSA)

Beyond identifying DE receptors, we sought to compute their potential downstream activation signaling. We postulate that such signaling cascades are evident in the cell’s transcriptomic profile, eventually converging at a downstream transcription factor.

Initially, transcription factors with enriched target genes in each cluster were identified. To counteract the inherent “zero inflated” data in single-cell analyses, we computed these parameters on a cluster-specific basis. Transcription factors with an FDR-adjusted p-value ≤ 0.05 were retained. We then determined a maximum flow from every receptor towards the enriched layer of transcription factors, utilizing a network of protein-protein interaction^28^.

To enhance our confidence in the identified pathways, the network was normalized. Each edge was weighted based on the mutual information (MI) between expression distributions in the cluster’s constituent genes. This was defined as:

1 $$I\left(X\!:Y\right)=\sum _{x}\sum _{y}{P}_{X,Y}\left(x,y\right)\cdot \log \left(\frac{{P}_{X,Y}\left(x,y\right)}{{P}_{X}(x)\cdot {P}_{Y}(y)}\right)$$

Where $(X,Y)$ is a pair of random variables, ${P}_{X,Y}(x,y)$ is their joint distribution and ${P}_{X}$, ${P}_{Y}$ the margin distribution of $X,Y$. To calculate the MI, we initially discretize the gene expression values. This was achieved by grouping the expression values into bins, with each bin containing expression data from 100 cells. For smaller clusters, we ensured a minimum of 10 bins per cluster. Using the MI between each pair of genes in the cluster, we marked the edge weight as follows:

2 $$\forall\, <\, {g}_{1},{g}_{2}\, >\, \in E\!:{weight}\left({g}_{1},{g}_{2}\right)=\frac{I\left({e}_{1},{e}_{2}\right)-\mathop{\min }\limits_{{ < g}_{i},{g}_{j} > }I({e}_{i},{e}_{j})}{\mathop{\max }\limits_{{ < g}_{i},{g}_{j} > }I({e}_{i},{e}_{j})-\mathop{\min }\limits_{{ < g}_{i},{g}_{j} > }I({e}_{i},{e}_{j})}$$

Here, $< {g}_{1},{g}_{2} >$ denotes an edge in the PPI network, and ${e}_{1},{e}_{2}$ are the expression vectors in the signal receiver cluster of the genes ${g}_{1},{g}_{2}$ respectively.

Upon creating this weighted network, a virtual ‘sink’ node was integrated. Each identified transcription factor was linked to this sink with an infinite edge weight. Within the resultant normalized network, each edge weight defines its flow capacity. Subsequently, Dinitz’s algorithm^30^ was applied to discern the maximum flow from receptors through the signaling pathway, concluding at the virtual sink. In our basic setting, the maximum flow is calculated separately for each receptor, resulting in a unique score for each receptor. This process aids in ranking the impact of various receptors in the interaction. Additionally, interFLOW can be configured in a multi-source mode, enabling simultaneous flow analysis from all receptors. Using this setting we can calculate the scores of downstream transcription factors (TFs), integrating cumulative signals from multiple receptors to a single TF.

To gauge the flow value’s significance, we employed a random, degree-preserving permutation on the signaling network. Each permutation involved edge shuffling 10x|E| times, with |E| representing the graph’s edge count. The switching algorithm^31^ was employed and max flow was calculated for each permutation. This allowed for an empirical statistical value representation of observed flow (post-FDR correction), compared against flows on randomized networks.

The integration of MI into our framework enables the identification of gene-gene relationships while also providing robustness against the inherent characteristics of scRNAseq data, zero-inflation and loss of correlation. Although MI has the potential to capture unrelated gene relationships, the use of the maximum flow algorithm in our framework adds a layer of specificity. For a high-weighted edge to significantly influence our predictions, it must form part of a clearly defined path from the source to the target. This requirement substantially reduces the likelihood of false positives arising from unrelated relationships.

### Generating a global interactions map

Finally, the interaction score between clusters (c1, c2) was computed for every cluster pair using the formula:

3 $${Score}\left({c}_{1},{c}_{2}\right)=\sum _{r\in {Receptors}}\sum _{L\in {Ligands}(r)}{DSA}\left(r\right)\cdot \bar{{e}_{1r}}\cdot \bar{{e}_{2l}}$$

Here, ${Ligands}(r)$ represents ligands in ${c}_{2}$ corresponding to receptor $r$, ${DSA}(r)$ denotes the receptor r’s flow score, while ${e}_{1r}$ and ${e}_{2l}$ are the normalized expressions of receptor $r$ in cluster ${c}_{1}$ and ligand l in cluster${c}_{2}$, respectively.

### Simulations

To capture the underlying assumption that activated pathways manifest co-expression patterns in the data we devised a simulation framework. This framework necessitated the capacity to simulate a correlated gene expression structure. To initiate this process, we leveraged the PPI network to randomly select pathways from a given set of $k$ transcription factors to a distinct subset of $w > k$ receptors. These randomly generated paths served as representative downstream activation pathways (DSPs). Subsequently, we proceeded to model latent gene expression representations using Multivariate Normal (MVN) distributions, denoting ${x}^{l} \sim {MVN}(\bar{0},\varSigma ),$ where $\bar{0}$ signifies the zero vector and $\varSigma$ represents the covariance matrix. In accordance with our design, we specified the covariance between any two genes, both$i$ and $j$, not part of the DSP $(i,j\notin {DSP})$ as $\mathrm{cov}(i,j) \sim N(0,\sigma )$. For genes within the true downstream activation pathway $(i,j\in {DSP})$, the covariance was defined as $\mathrm{cov}(i,j) \sim N(\mu ,\sigma )$, where $\mu$ quantifies the degree of co-expression within the downstream activation pathway. To control for the potential of unrelated genes to exhibit high correlation by chance, we tested the performance of our framework under high noise conditions by selecting a relatively high σ value of 0.1. This selection resulted in approximately 5% of false edges achieving a correlation of more than 0.2 (Supplementary Fig. 2), thereby introducing negative edges with medium-to-high correlation into the framework. Recognizing that authentic gene expression data does not conform to a normal distribution but can be modeled more appropriately using the Zero-Inflated Negative Binomial (ZINB) distribution, we sought a transformation that would preserve the inherent correlation structure. Our approach involved applying the cumulative distribution function (CDF) of the standard normal distribution to our latent gene expression values, yielding a set of correlated uniform deviates$.$ subsequently, we employed the quantile function of the Negative Binomial distribution (with a randomly sampled mean) and added random dropout to derive the final gene expression values for the given cluster. After implementing these transformations, we achieve our final simulated dataset. In this dataset, the correlation structure is preserved in a nonlinear manner. Interestingly, the Pearson correlation within the DSP loses significance, while the Spearman correlation remains significant. This configuration better captures the attributes of real single-cell data.

Specific dimensions of our simulated data (number of genes that were simulated) were based on the union of our prior knowledge databases. Genes not present in any of these databases do not influence the prediction of interFLOW and, thus should be excluded from the simulation. It is also important to note that the correlation structure between different cell populations has minimal impact on our model’s performance. This is because the weighting of the PPI network, which is calculated using the co-expression within the signal-receiving cluster alone, as this network aims to model only this cluster’s intracellular signaling pathway. Consequently, our prepose simulation framework does not account for this correlation structure.

## Materials and Methods IHC

### Primary Immunostaining antibodies

Rabbit anti-mouse IBA1 (Cat. No. NBP2-19019; Lot. No. 43796; Dilution: 1:200), rabbit anti-mouse CCR1 (Cat. No. NB100-56334SS; Lot. No. B-03; Dilution: 1:200), rat anti-mouse F4/80 (Cat. No. NBP1-60140; Lot. No. 28094M1219-A; Dilution: 1:100), and rabbit anti-mouse NRF2 (Cat. No. NBP1-32822; Lot. No. 44749; Dilution: 1:100) were purchased from Novus (Colorado, USA). Rat anti-mouse CD4 (Cat. No. 14-9766-B2; Lot. No. 2526300; Dilution: 1:100), mouse anti-mouse CD68 (Cat. No. MA1-7631; Lot. No. ZA4163501; Dilution: 1:50), and rabbit anti-mouse STAT5 (Cat. No. PA5-40241; Lot. No. YH4029074; Dilution: 1:150) were purchased from Invitrogen (Massachusetts, USA). Rabbit anti-mouse CD28 (Cat. No. ab243228; Lot. No. 1003834-6; Dilution: 1:50), rabbit anti-mouse CD86 (Cat. No. ab119857; Lot. No. 1004179-2; Dilution: 1:50), rabbit anti-mouse RelA/p65 (Cat. No. ab32536; Lot. No. 1006853-3; Dilution: 1:100), and rabbit anti-mouse pSTAT5 (Cat. No. AB32364; Lot. No. 1020646-16; Dilution: 1:50) were purchased from Abcam (Cambridge, UK).

### Secondary immunostaining antibodies

Goat anti-rat Alexa Fluor® 647 (Cat. No. ab150159; Lot. No. 1005431-5; Dilution: 1:250), Goat anti-rabbit Alexa Fluor® 568 (Cat. No. ab175471; Lot. No. 1044159-1; Dilution: 1:250), and Goat anti-rabbit Alexa Fluor® 488 (Cat. No. ab150113; Lot. No. 1001014621; Dilution: 1:250) were purchased from Abcam (Cambridge, UK). Goat anti-mouse Alexa Fluor® 647 (Cat. No. 115-605-166; Lot. No. 161227; Dilution: 1:250) was purchased from Jackson ImmunoResearch (Pennsylvania, USA).

### Immunostaining

OCT blocks of brain tumors from GL261 glioblastoma-bearing mice (shSELP and shNC) were cut into 5 µm thick sections. Immunostaining was performed using the BOND RX Multiplex IHC AutoStainer (Leica). Double immunostaining sections were stained for F4/80 using rat anti-mouse together with either rabbit anti-mouse RelA/p65, rabbit anti-mouse NRF2, or rabbit anti-mouse CCR1. CD4 using rat anti-mouse together with either rabbit anti-mouse CD28 or rabbit anti-mouse STAT5. Alexa Fluor® 647 goat anti-rat and Alexa Fluor® 568 goat anti-rabbit were used as secondary antibodies. Triple sections were stained with CD4 using mouse anti-mouse together with both rat anti-mouse F4/80 and rabbit anti-mouse pSTAT5. Alexa Fluor® 647 goat anti-mouse, Alexa Fluor® 568 goat anti-rat, and Alexa Fluor® 488 goat anti-rabbit were used as secondary antibodies. Prior to antibody incubation, slides were incubated with 10% normal goat serum in 1x Tris-buffered saline, 0.1% Tween-20®, for 30 min to block non-specific binding sites. Slides were incubated with the primary antibody for 1 h, then washed and incubated for another 1 h with the secondary antibody. The slides were stained with Hoechst for nuclei detection. Then, ProLong® Gold mounting was applied on the slides prior to being covered with coverslips. The stained slides were captured using a Cytation C10 confocal imaging reader (spinning disk 60 µm) (Agilent, California, USA) and analyzed using the Gen5 program (Agilent, California, USA). Nuclear expression of NRF2 was analyzed by measuring the pixel area of NRF2 within the nucleus (DAPI). The distance matrix was analyzed using the MACSiQView app (Miltenyi, Germany).

### scRNA and Prior Information Dataset

1. **Simulated Data:** This framework was developed and utilized to generate multiple simulated scRNAseq datasets. These datasets comprise signal-receiving and signal-sending clusters, with each cluster containing 1000 cells across 6347 genes. They were employed to evaluate the performance of interFLOW across various tasks.
2. **Yeini et al. Glioblastoma Multiforme (GBM) Mouse Model**: As described in^4^, this dataset includes 9175 cells and 18,531 genes from both control and treatment GL261 tumor model samples (treatment involved inhibition of P-selectin). It served for further validation of our framework and for conducting an in-depth analysis of the crosstalk between tumor infiltrating CD4 T cells and macrophages.
3. **Glioma Brain Tumor Mouse Model:** This dataset, referenced in ^21^, comprises 18,023 cells across 15,749 genes. It was utilized as an additional data source for validation and benchmarking, demonstrating the robustness of the analysis.
4. **Protein-Protein Interaction Network:** Detailed in^28^ the size of the network used varies depending on the expressed genes within each cell cluster, according to our datasets. The average number of genes was 4054, with a standard deviation of 164 genes. The average number of edges was 36,504, with a standard deviation of 1452.
5. **Ligand-Receptor Database:** As outlined in^9^ this database contains 643 ligands and 829 receptors, encompassing 12,156 interactions.

### Reporting summary

Further information on research design is available in the Nature Research Reporting Summary linked to this article.

### Supplementary information

Supplemental Material

Reporting summary

## Data Availability

All datasets analyzed in this manuscript are publicly available and have been documented under the “scRNA and Prior Information Dataset” section.

## Code availability

interFLOW is available for download via the git repository github.com/madilabcode/interFLOW, including all the necessary files and conda environment, along with an explanation of basic usage of the framework.
