# Supplementary material for: interFLOW: maximum flow framework for the identification of factors mediating the signaling convergence of multiple receptors
Source: NPJ Syst Biol Appl. 2024 Jun 10;10:66. doi: 10.1038/s41540-024-00391-z (PMC11164912; doi:10.1038/s41540-024-00391-z)
Supplement: Supplementary file 1 — Supplemental Material [file 41540_2024_391_MOESM1_ESM.pdf]

Supplementary materials for interFLOW: maximum flow framework for the identification of factors mediating the signaling convergence of multiple receptors

Ron Sheinin[1], Koren Salomon[2], Eilam Yeini[2], Shai Dulberg[3], Ayelet Kaminitz[3], Ronit Satchi-Fainaro[2,4], Roded Sharan[1, *] and Asaf Madi[3, *]

[1]Blavatnik School of Computer Science, Tel Aviv University, Tel Aviv 6997801, Israel.

[2]Department of Physiology and Pharmacology, Faculty of Medicine, Tel Aviv University, Tel Aviv 6997801, Israel.

[3]Department of Pathology, Faculty of Medicine, Tel Aviv University, Tel Aviv 6997801, Israel.

[4]Sagol School of Neurosciences, Tel Aviv University, Tel Aviv 6997801, Israel

[*]Equal contribution

Cell Population	Receptors
CD8 T Cells	Trac, Trbc, Cd8a, Cd8a, Cd8b, Pdcd1, Ctla4, Itgal, Itgal2, Cd28, Klra, Klrk1, Cd27, Tnfrsf4, Tnfrsf9, Havcr2, Icos, Cd44, Ccr7
CD4 T Cells	Trac, Trbc, Cd4, Cd28, Pdcd1, Ctla4, Icos, Itgal, Itgal2, Cxcr5, Cd69, Tnfrsf4, Tnfrsf9, Havcr2, Icos, Ccr7, Il2ra.
Macrophages	Tlr4, Tlr2, Cd14, Fcgr3, Fcgr1, H2-Aa, H2-Ab1, Itgam, Itgax, Ccr2, Cx3cr1, Clec7a, Cd36, Msr1, Marco
Microglia	Tlr4, Tlr2, Itgam, Ptprc, Cx3cr1, P2ry12, Term2, Cd200r1, Ager, H2-Aa, H2-Ab1,Fcgr3, Fcgr1. Itgal, Itgb2, Itgam, Itgax, Cd36
Neg	Mb1, Cd79a, Cd79b, Cd19, Ms4a1, Cr2, Cd40, Cd22, Cd72, Tnfrsf13c, Tnfrsf17, Tnfrsf13b, Kit, Fpr1, C3ar1, C5ae1, Tpsab1, Cap3, Hdc, Eger, Erbb2, Kdr, Cd47, Fgfr1, Fgfr2, Fgfr3,, Fgfr4, Muc1

Supplementary Table 1: Cell type-specific receptors

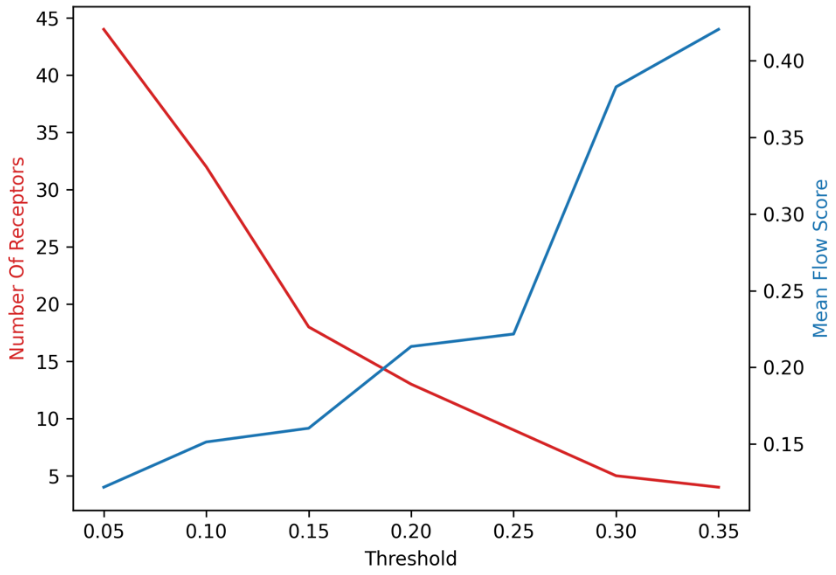

Supplementary Figure 1. Threshold optimization. This figure illustrates the number of receptors retrieved from the Ligand-Receptor DE (Differentially Expressed) filtering process at each threshold level, along with their mean flow score. We aim to include more receptors in the downstream analysis and to rank these receptors using the DSA (Downstream Signaling Activity) score. Here, depicting the interaction of CD8 cells in the GBM (Glioblastoma Multiforme) dataset, we observe that increasing the threshold from 0.1 to 0.2 significantly reduces the number of receptors, while the mean flow score only increases from 0.15 to 0.2. Therefore, we opt for the more lenient threshold of 0.1 to include a broader array of receptors for detailed analysis.

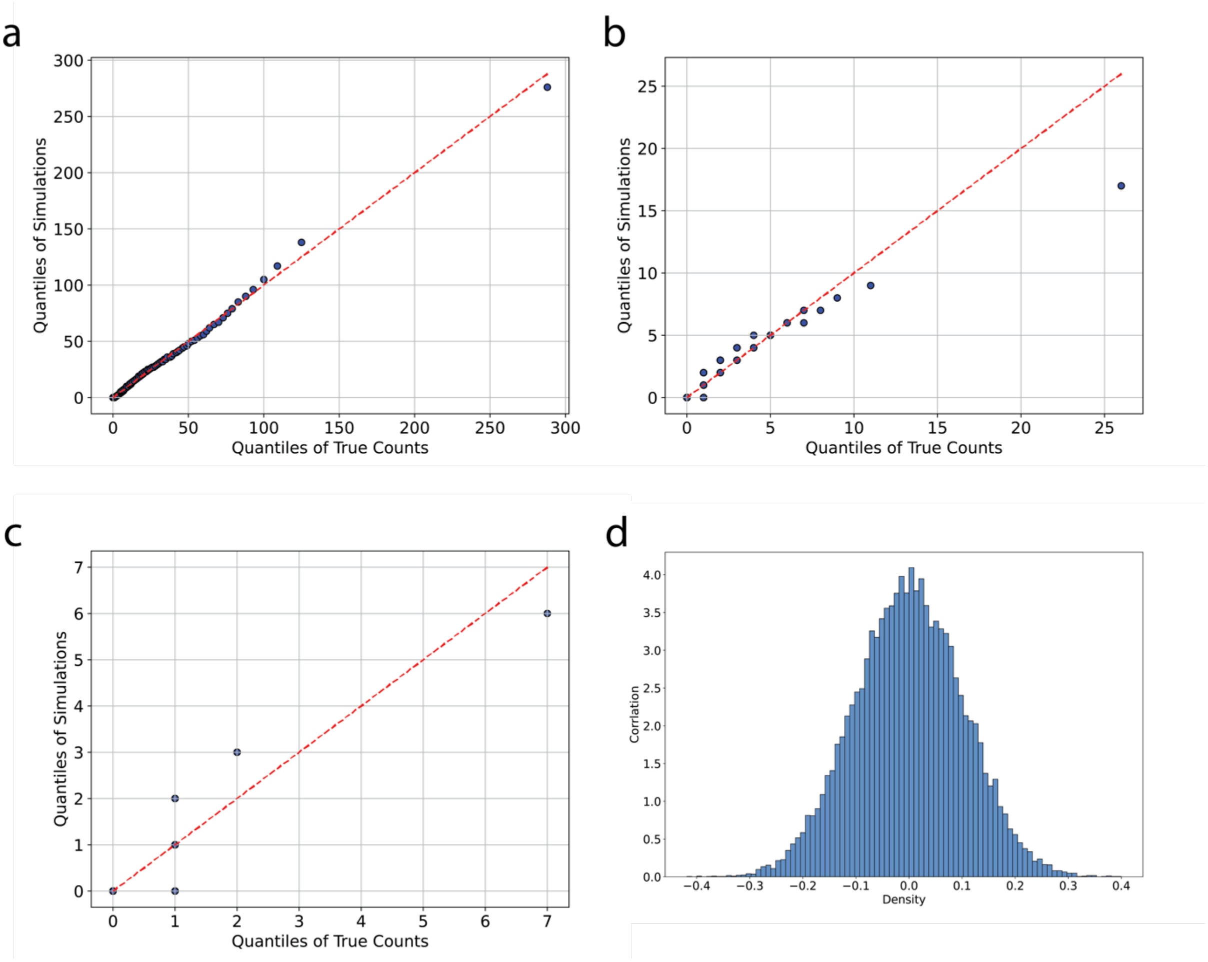

Supplementary Figure 2. Simulation. (a-c) Q-Q plots of simulated genes and the true expression. (a) Low dropout gene (Cd3g) with dropout of 0.1. (b) Medium dropout gene (Cdk4) with a dropout of 0.5. (c) High dropout gene (Tifa) with a dropout of 0.8. (d) distribution of random correlation of false edges in our simulations.

Validation of up-regulated receptors

We checked if receptors that are up-regulated in a given cluster are associated with high flow values defined by our generated network. Thus, not only is the receptor itself upregulated but also its downstream signaling pathway.

To this end, for each cluster, we identified a set of receptors that are upregulated relative to all other clusters in the data set. We then defined an equally balanced list of receptors that are upregulated in the cluster and receptors which are not upregulated. For each receptor, we calculated the max flow value, and used these values to calculate ROCAUC of the classification of upregulated receptors (**Supp. Fig. 3**).

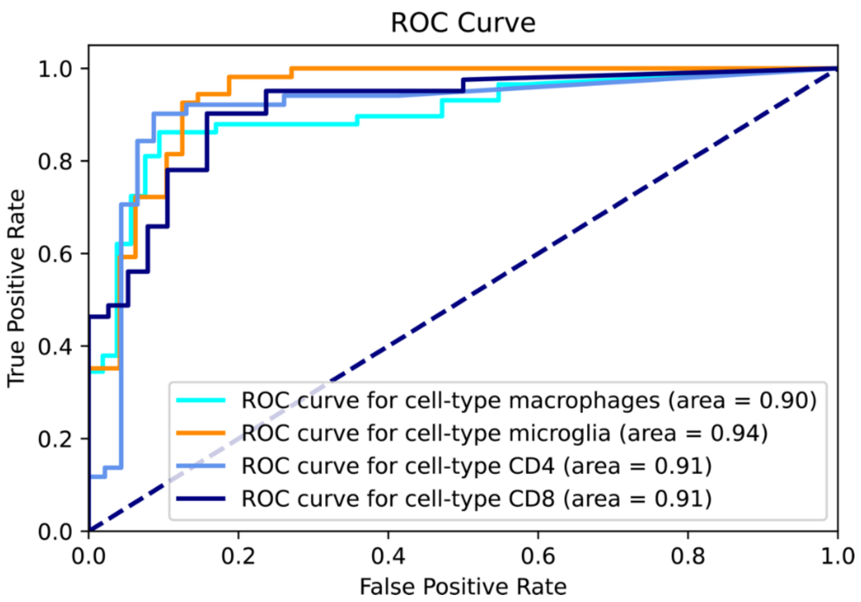

Supplementary Figure 3. Validation of receptors detection. ROC curve analysis of upregulated receptors by flow shows that flow values are significantly associated with upregulated receptors.

NicheNet MSigDB comparison

We run our MSigDB validation for the downstream signal return from NicheNet. First, the activation score of each gene was calculated for each ligand returned by the method. Next, we summed the activation score of each gene over all the identified ligands, then we chose the top n genes (where n is the number of genes returned by interFLOW) with the highest score to the enrichment analysis; the results are presented in **Supp. Fig. 4.**

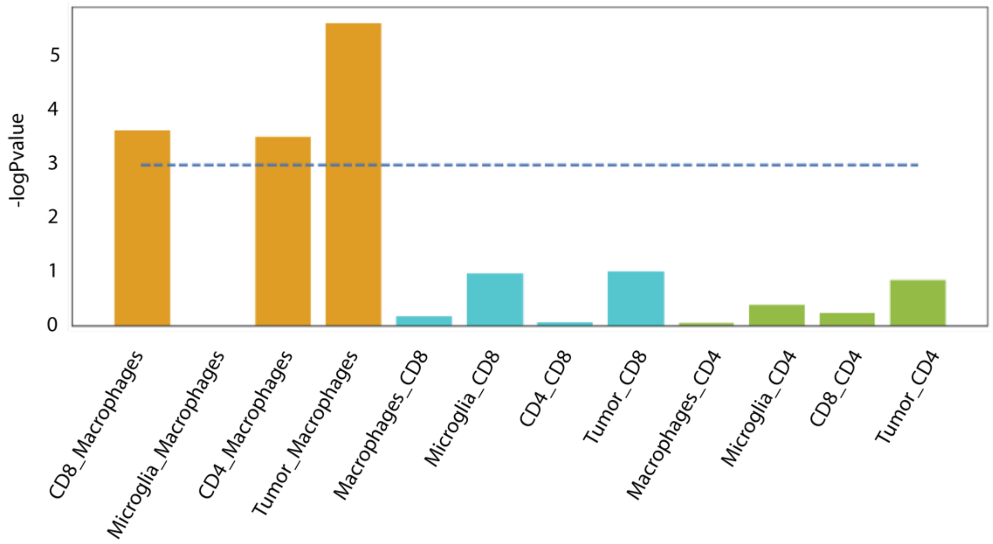

Supplementary Figure 4. Validation of NicheNet against MSigDB C7 immunological signature database.

Comparison to CellChat method

We compared our results to the recently published CellChat algorithm. As discussed previously, comparing the identification processes of cluster-specific receptor-ligand pairs using different tools is a non-trivial task. CellChat provides cell-cell communication on a global scale, whereas we were aiming to see if the same biological trends can be observed using the CellChat toolkit, as we observed by interFLOW. We generated all-to-all interactions using CellChat, and compared the Immune interactions to the non-immune interactions score. CellChat mean score of the immune interaction was significantly higher than the non-immune interaction (p-value = 0.03), which is thus in agreement with the interFLOW method (**Supp Fig. 5**).

As we cannot compare the methods to a “gold standard” data set, we next compared the relationship between the methods ’scores. To this end, we compared interFLOW to the score of each receptor. We discovered a significant correlation of 0.4 between our flow scores and CellChat communication probability.

Furthermore, we compared our flow permutation score between receptors that were common to both methods and receptors that were not. We found a significant difference in the mean score value between the two groups (p-value = 0.006), and ROC AUC of 0.7.

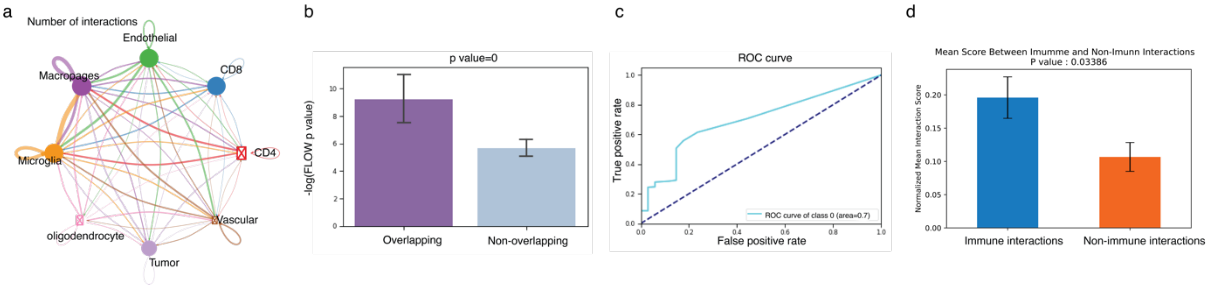

Supplementary Figure 5. Comparison to the CellChat method. (a) CellChat global interaction map. (b) Mean score of overlapping and non-overlapping interactions between the methods. (c) ROC AUC results for the overlapping interactions by the interFLOW score. (d) Normalized mean interactions score of immune cell types compared to interactions between non immune cell types, p value was calculated using t test statistic.

Benchmark on Glioma Brain Model

To check the robustness of our framework, we ran our benchmark pipeline on the published single-cell RNA seq dataset of a mouse brain glioma model; a summary of the results is shown in **Supplementary Figure 6**.

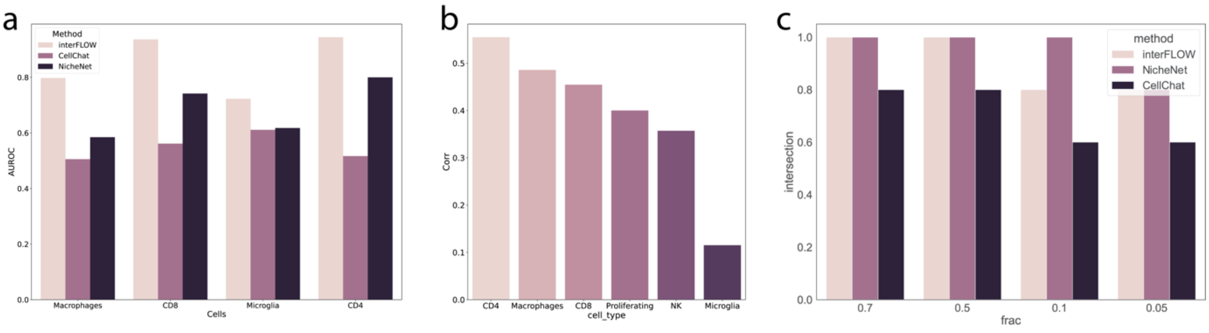

Supplementary Figure 6. Validation on Lymph node dataset. (a) ROC analysis of identify cell specific receptors. (b) Spearman correlation between TFs interFLOW score and target enrichment score per cell type. (c) Robustness analysis of the methods prediction.

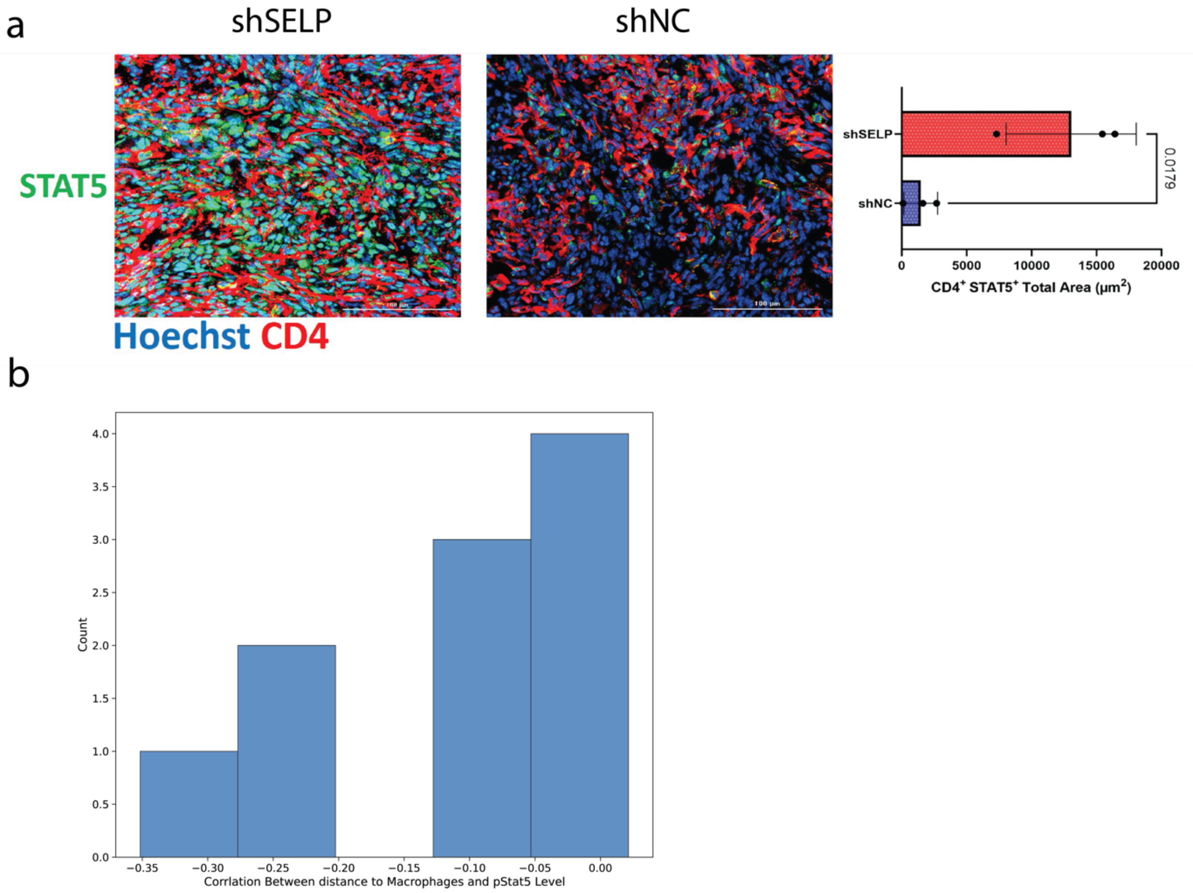

Supplementary Figure 7. Silencing SELP in GL261 glioblastoma tumors alters the macrophages 'co-stimulation of CD4 T cells. (a) Immunostaining analysis of GL261 glioblastoma tumors showed increased expression of STAT5 in CD4 T cells in SELP knockdown GL261 tumors (shSELP) compared to the negative control (shNC). Data are represented as the mean ±s.d. Each dot (N=3) indicates the average of five fields in the tissue. The analysis was carried out using an unpaired two-tailed T-test. (b) Spearman correlation distribution between expression levels of pSTAT5 on CD4+ T cells and their minimal proximity to macrophages in the different slides.

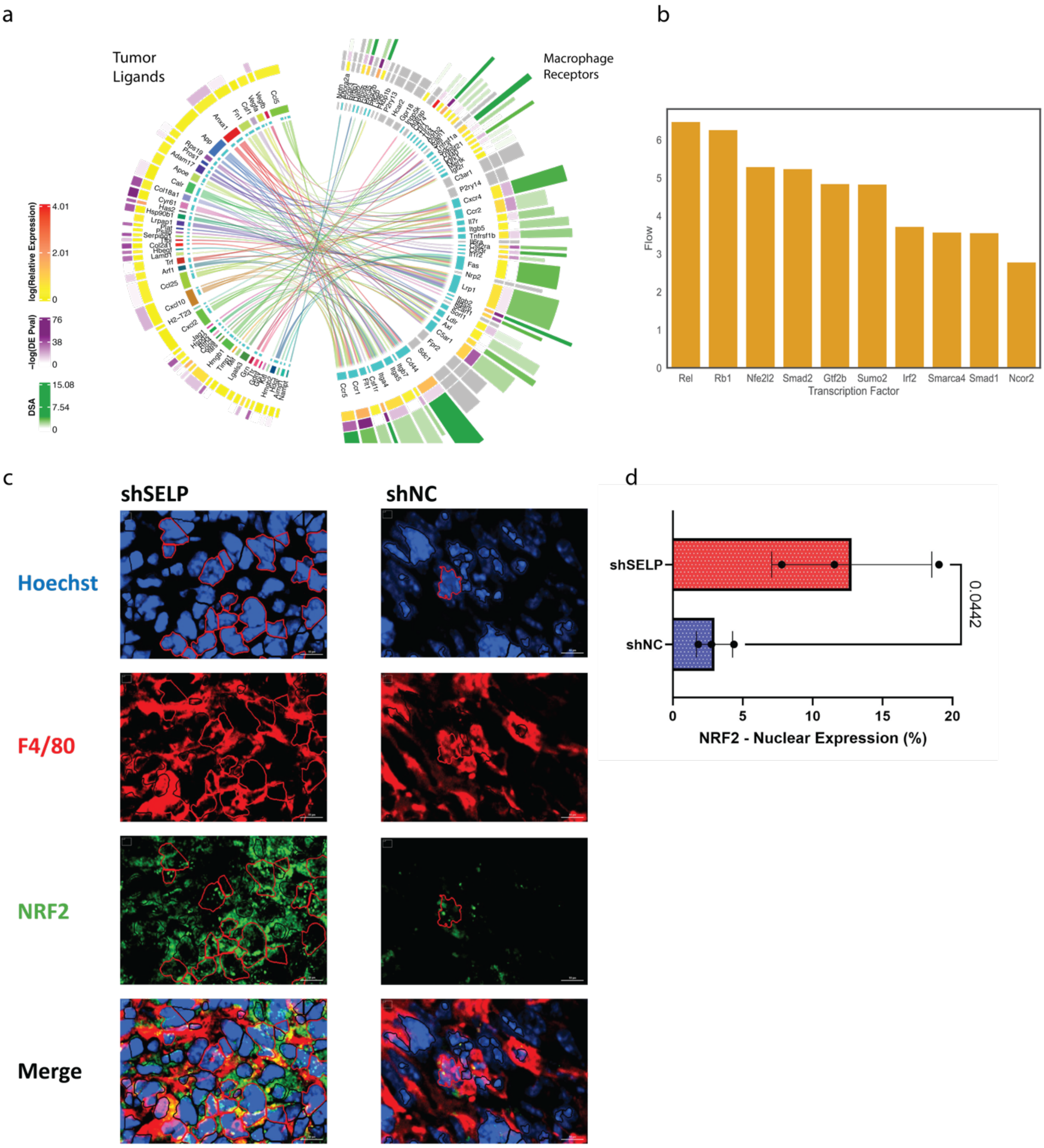

Supplementary Figure 8. Cross talk between tumor and macrophage clusters. (a) Ligand – Receptor circus plot. (b) Transcription factor's important score. (c) Immunostaining analysis of GL261 glioblastoma tumors showed increased expression of nuclear NRF2 (Nfe2l2) in macrophages in SELP-knockdown GL261 tumors (shSELP) compared to the negative control (shNC). (d) Data summary represented as the mean ± s.d. Each dot (N=3) indicates the average of five fields in the tissue. The analysis was carried out using an unpaired two-tailed T-test.

Global interaction quantification

In the global analysis of the current dataset, we expect to find stronger interactions to or from immune compared to non-immune clusters. To quantify such a trend, we calculated the mean interaction score

of immune-related interactions (at least one of the clusters in the interaction represents immune cell type), to the mean score of non-immune interaction (**Supp. Fig. 9**). As expected, the immune interaction score was indeed stronger.

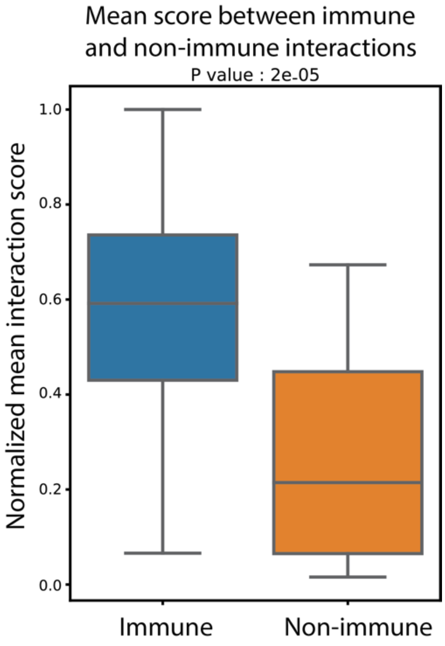

Supplementary Figure 9. Validation of global immune and non-immune interactions and method robustness. Normalized mean interaction score of immune cell types compared to interactions between non-immune cell types, p-value was calculated using t-test statistic.
